# Selective uptake and sensing of nitrate in poly(3,4-ethylenedioxythiophene)

**DOI:** 10.1038/s41598-017-16939-5

**Published:** 2017-11-29

**Authors:** Sam Rudd, Michael Dalton, Peter Buss, Amanda Treijs, Michael Portmann, Nick Ktoris, Drew Evans

**Affiliations:** 1 Future Industries Institute, University of South Australia, Mawson Lakes, South Australia 5095 Australia; 2 Sentek Pty Ltd, Stepney, South Australia 5069 Australia

## Abstract

Nitrogen (N) as a nutrient, in the form of nitrate (NO_3_^−^), is essential for plant growth. Chemical fertilizers are used to increase crop yields, but overuse can lead to forms of environmental pollution necessitating methods to detect and monitor the level of NO_3_^−^ *in-situ* in agricultural soils. Herein we report for the first time the NO_3_^−^ selectivity of the inherently conducting polymer poly (3,4-ethylenedioxythiophene) (PEDOT). This selectivity occurs when PEDOT thin films are exposed to an aqueous environment containing not only NO_3_^−^, but a mixture of other ions present in concentrations (ppm) typical of real agricultural soil. The PEDOT sensitivity to absorb NO_3_^−^ from solution is determined to be <1 ppm.

## Introduction

In order to enhance the effectiveness of the soil and improve quality and yield in agricultural production, fertilizers and chemical materials are typically utilised to assist in the global need for food, feed, fuel and fibre^1,2^. With the continued use of chemicals in modern industries and agriculture, detection and monitoring of anion contamination is increasingly important. For instance, nitrate (NO_3_^−^) levels above 10 mg/L (10 ppm) in groundwater, originating from nitrogen-based fertilizers, have been linked to health issues for humans^3^.

The promising features of conducting polymers (CPs) have attracted significant attention from scientists aiming to improve the conductivity of CPs as synthetic metals^4^. Among the known CPs, poly(3,4-ethylenedioxythiophene) (PEDOT) has been considered a desirable material in a variety of applications including solar cells^5^, organic light emitting devices^6^ and medical devices^7^ owing to its unique characteristics of excellent conduction and electro-optical properties. Developments in the synthesis of CPs have shown that PEDOT becomes highly conductive when doped and/or with certain post-treatment^8^. In principle, the pristine PEDOT is obtained indirectly via de-doping, hence electrochemical techniques such as cyclic voltammetry are used to discharge or recharge CPs in the presence of an electrolyte (salt solution containing anions)^9^. As a response to electrochemical oxidation or reduction, the electrical and optical properties of PEDOT change significantly due to the doping or de-doping process (anion uptake or release)^10,11^. This phenomenon makes PEDOT an ideal substance for controlled drug release applications^12^, however anion uptake of this CP can be employed in other advanced applications such as sensors. For instance, Zampetti *et al*. reported a highly sensitive sensor using an ultra-thin layer of PEDOT to monitor NO and NO_2_ gases in breath and eventually to monitor asthma^13^. In another study Luo and co-workers used PEDOT as a matrix of a nanocomposite to create an electrochemical sensor for detection of nitrite^14^. Gokhale *et al*. immobilised nitrate reductase enzymes within PEDOT nanowires, where the enzymes reduce nitrate to nitrite, and the resultant electrons are transferred via methyl viologens in solution to the PEDOT bioelectronic sensor^15^.

In recent years, various materials and methods have been trialled for the purpose of developing a nitrate sensor for agricultural purposes^16–19^. Coupling LED light sources in a spectrophotometric analysis allows for colour-developing agents to be mixed with extracted water samples to yield colour intensity vs concentration curves^20^. These calibration curves then allow for determination of anion content within the extracted water. Weber and co-workers utilised an impedance technique in a two electrode flow cell arrangement with extracted water from soil added to a known soil mix^21^. The dielectric constant of the known soil mix when dry is compared to the same mix when wetted with different total salt content. Without providing details of how, this process is reported to be adaptable to specifically sense NO_3_^−^. More recently from the same research group, Ali *et al*. reported an electrochemical sensor comprising a working electrode of electrospun PEDOT nanofibers decorated with graphene oxide on gold substrates^22^. Enzymes to convert nitrate to nitrite are immobilised on the graphene oxide, hence rendering an electrochemical reaction that can be determined via electrochemical impedance spectroscopy. This method requires a nitrate sensitive working electrode, plus a counter and reference electrode. Further, Higson and co-workers, in their excellent review of anion selective sensors, highlight that doping CPs with anion-recognition sites presents an ideal scenario for simple detection of specific anions^23^. Many of the anion sensing-CP studies to date have focused on polypyrrole^24,25^ and its derivatives^26^ using electrochemical processes for detection. In the present work, we fabricated simple PEDOT-Tosylate thin films via Vapour Phase Polymerisation (VPP)^27^, electrochemically de-doped the PEDOT to remove most of the Tosylate, then selectively re-doped it with NO_3_^−^ directly from a mixed ion solution extracted from agricultural soil. This re-doping was done using concentrations of NO_3_^−^ in the ppm range, without any external electrical stimulus, nor with the need for engineering anion recognition sites. Selectivity for NO_3_^−^ was observed to be inherent for VPP PEDOT.

## Results and Discussion

**Figure 1:**
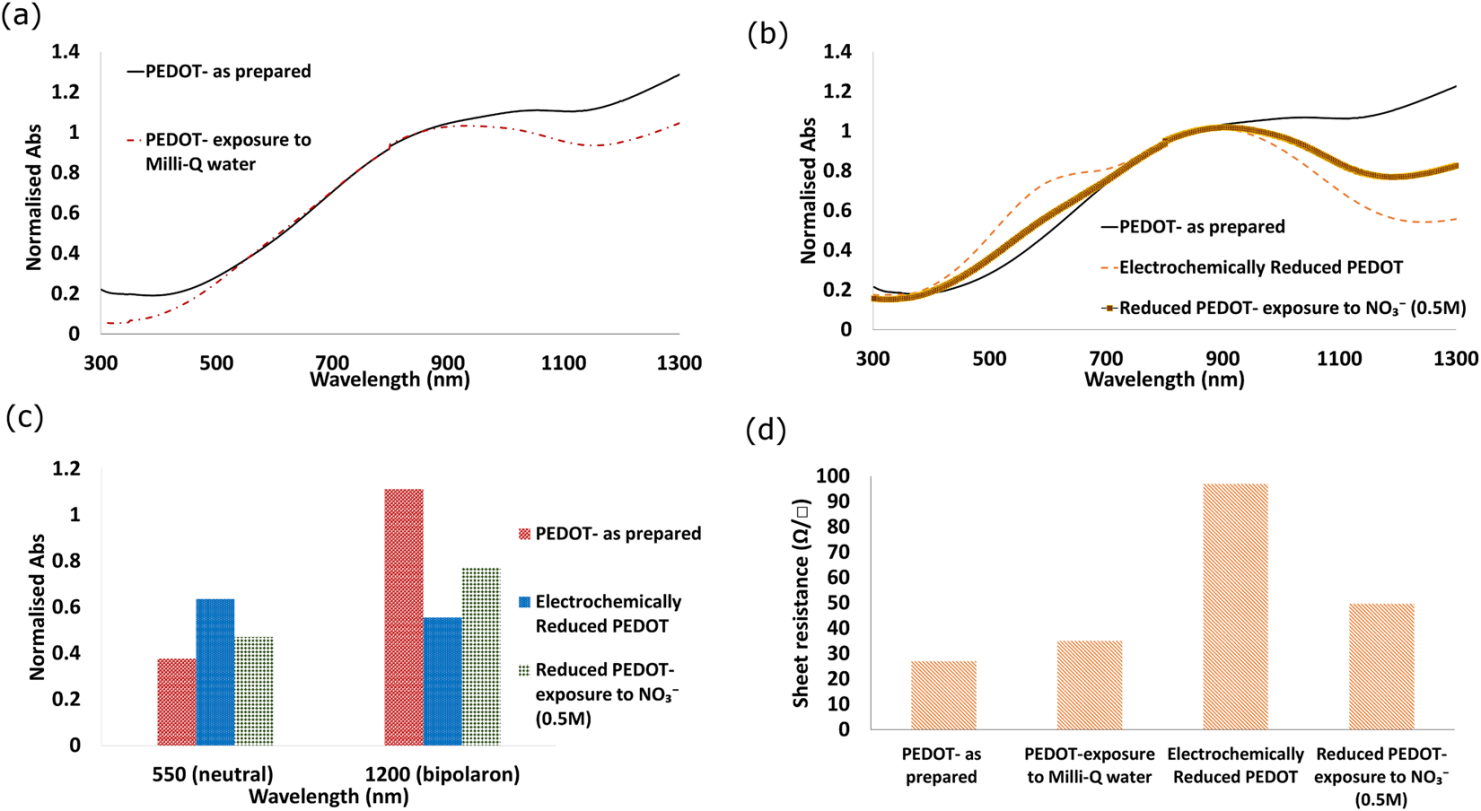
Properties of reduced PEDOT exposed to NO_3_−. Absorption spectra of (**a**) PEDOT as-prepared and after reduction by Milli-Q water, (**b**) electrochemically reduced PEDOT before and after exposure to NO_3_^−^ (**c**) neutral and bipolaron of PEDOT- as prepared, electrochemically reduced and reduced PEDOT after exposure to NO_3_^−^, and (**d**) measured sheet resistance (Ω/□) of PEDOT before and after treatment.

The initial electrochemical reduction process leads to the sheet resistance (R_s_) of the PEDOT increasing - rendering the PEDOT lower in conductivity and higher in opacity, due to the decreased number of anions within^28^. Interestingly, the removal of anions from PEDOT can also be observed in the absence of any electrical stimuli. As-prepared PEDOT immersed in Milli-Q water (no Tosylate in solution) displayed an increase in optical opacity in line with anion removal and PEDOT reduction (Fig. 1a). When (electrochemically) reduced PEDOT is exposed to electrolyte containing NO_3_^−^ it appears more optically transparent to the eye, and is verified by UV-Vis spectroscopy (Fig. 1b). This occurs without the application of any external electric potential to drive electrochemical insertion of the anions, indicating the movement of anions in and out of the PEDOT occurs via diffusion. The diffusion is hypothesised to arise from the chemical potential difference between PEDOT and electrolyte with respect to the different anion concentration in each phase (PEDOT vs electrolyte).

Figure 1c and d follow the changes in optical and electrical properties of the PEDOT through the various steps. The as-prepared PEDOT has the lowest R_s_, which increases slightly upon exposure to Milli-Q water, and significantly when electrochemically reduced. In both cases anions are being released from the PEDOT. When the reduced PEDOT is exposed to the nitrate solution R_s_ decreases. This highlights the re-uptake of anions into the PEDOT film, thus doping or oxidising the PEDOT. The chemical analysis of the polymer film via X-ray photoelectron spectroscopy (XPS) confirmed the anion uptake by reduced PEDOT within this process (Figure S1).

**Figure 2:**
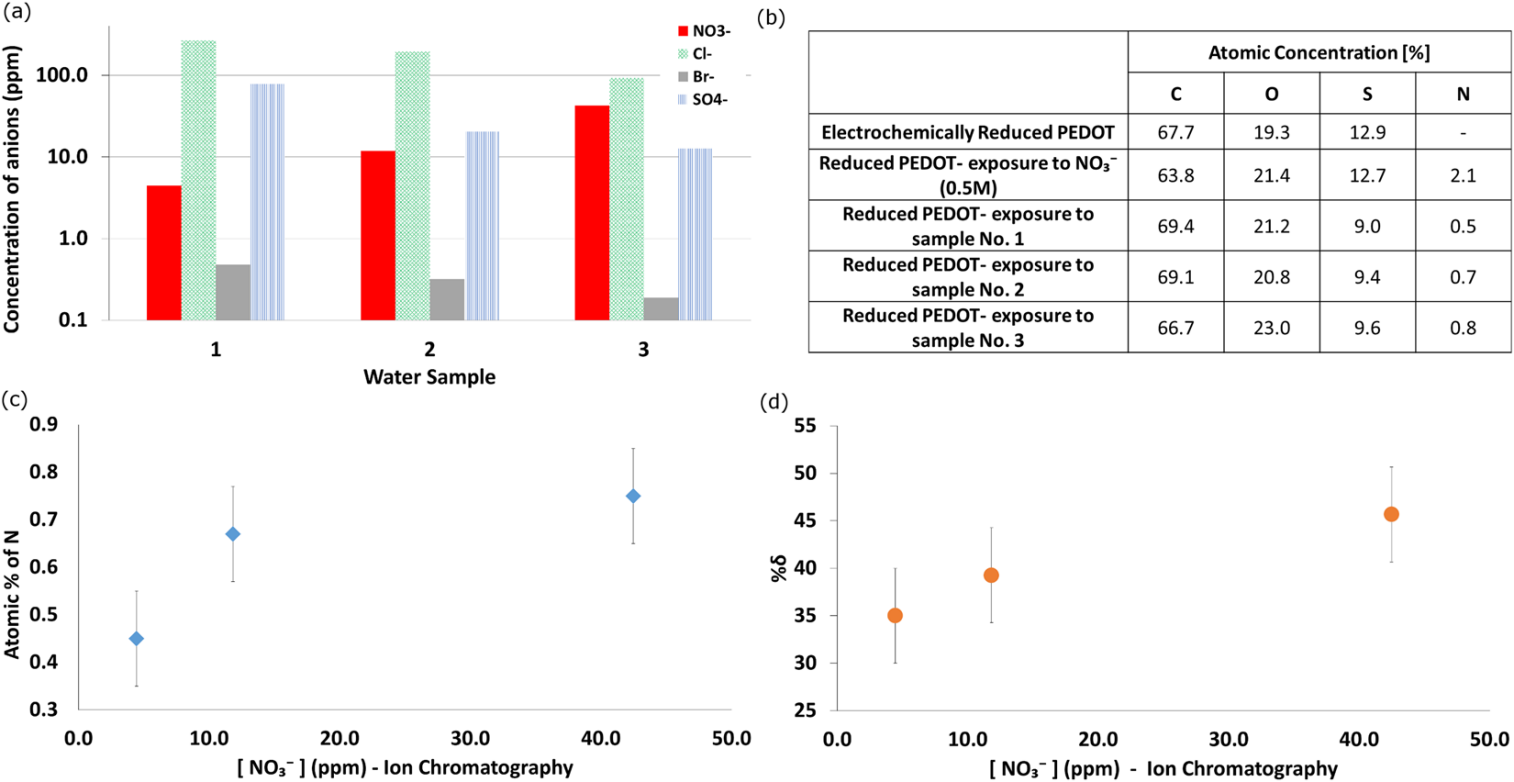
Exposure of reduced PEDOT to water extracted from agricultural soil. (**a**) Ion chromatography (IC) analysis of anions (see legend) present in the water samples (1 to 3) extracted from the field, (**b**) chemical analysis of treated PEDOTs using X-ray photoelectron spectroscopy (XPS), (**c**) atomic percentage of N absorbed by reduced PEDOT, and (**d**) calculated change of PEDOT’s sheet resistance after exposure to the extracted water samples 1 through 3.

The ion chromatography (IC) analysis of real water extracted from an agricultural field showed that in practice there is a mixture of ions in low concentrations (Fig. 2a). In fact, while there is a variation in NO_3_^−^ concentration, both the Cl^−^ and SO_4_^−^ concentrations are significant and Br^−^ is detectable. The use of three distinct samples in the current study is in contrast with other studies that rely on one sample diluted to various NO_3_^−^ concentrations^22^. Dilutions such as these not only change the NO_3_^−^ concentration but also the background ion content. Owing to the fact that PEDOT takes up anions from the surrounding aqueous environment, the performance and sensitivity to specific ions was also evaluated. The chemical analysis of the PEDOT films, using XPS, revealed that only NO_3_^−^ (in varying concentrations) was absorbed by PEDOT (Figs 2b and S2). The XPS spectra showed an absence of Cl^−^, SO_4_^−^ or Br^−^ (Figure S4 presents analysis of the S 2p fine scan). Comparing the XPS data for the atomic percentage of N with the NO_3_^−^ concentration in solution from IC shows that the uptake of N is related to the concentration of NO_3_^−^ in solution (Fig. 2c). The error bars in Fig. 2c and d represent the measurement errors in determining the atomic percentage and relative change in conductivity. Samples were found to be within these measurement errors.

The UV-Vis-NIR spectrophotometer was used to investigate the oxidation of PEDOT as a result of taking up anions (Figure S3). Although providing confirmation of the sensitivity of PEDOT to low concentration of anions it appears that the optical technique is not sensitive enough to correlate the oxidation level of PEDOT to anion concentration, as all spectra of treated films were almost the same. However, the comparison of the change in sheet resistance of treated PEDOT films illustrates a correlation with the concentration of NO_3_^−^ in solution (Fig. 2d).

**Figure 3:**
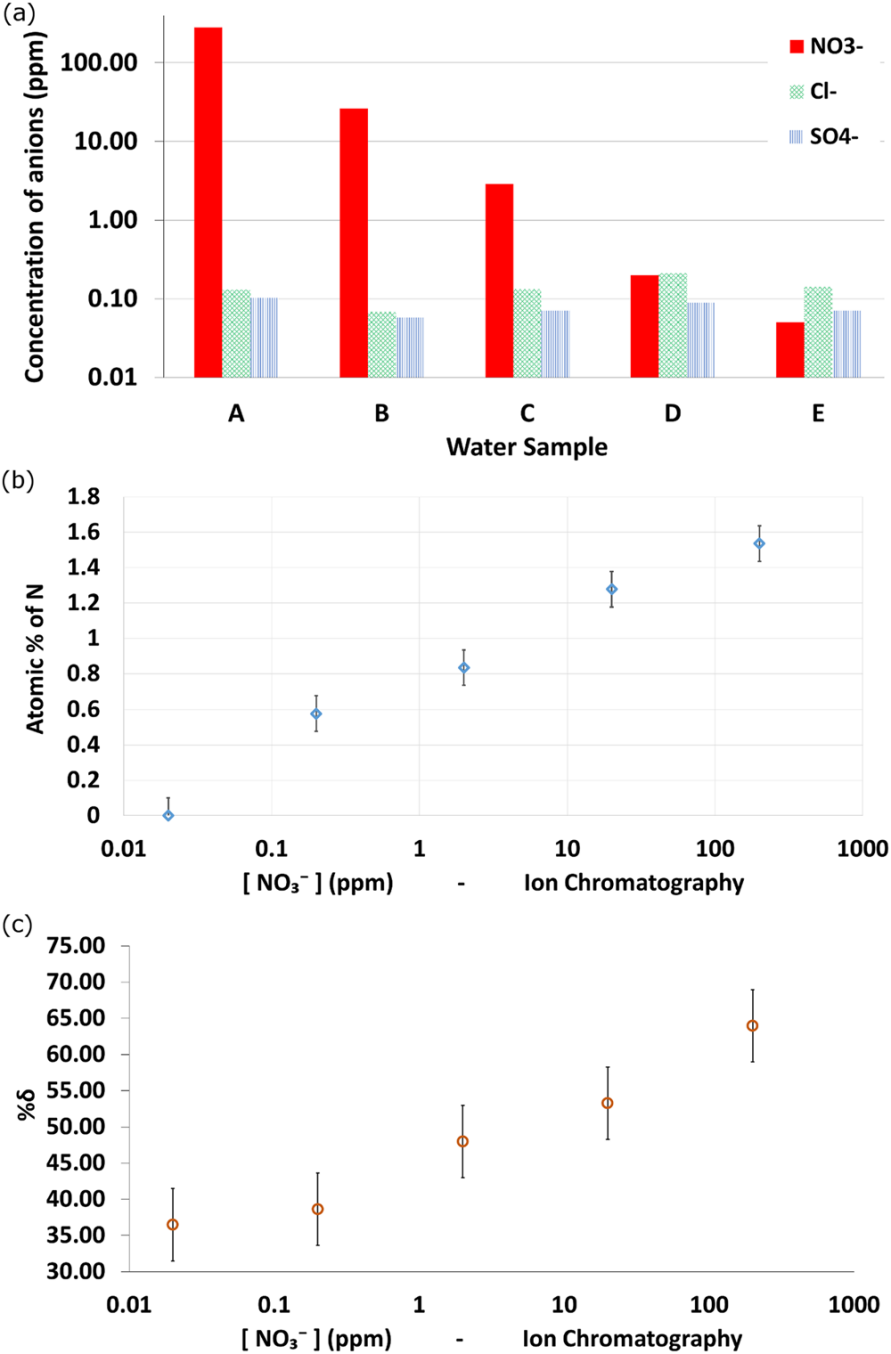
Uptake of differing concentrations of NO_3_− by reduced PEDOT. (**a**) Ion Chromatography analysis of anion content in samples A through E, showing decreasing [NO_3_^−^] and background [Cl^−^] and [SO_4_^−^], (**b**) atomic percentage of N taken up by the reduced PEDOT and (**c**) calculated change of PEDOT’s sheet resistance, after exposure to different [NO_3_^−^] solutions (log scale).

The correlation between N present and the change in electrical properties in the exposed PEDOT with the NO_3_^−^ concentration in solution was tested over a wider range of NO_3_^−^ concentrations (<0.05 up to 280 ppm). The chemical analysis of these solutions is provided in Fig. 3a (IC analysis). NO_3_^−^ is the major component of solutions A, B and C, whereas in D and E, Cl^−^, SO_4_^−^ and NO_3_^−^ all appear in concentrations <1 ppm. XPS analysis of the corresponding exposed PEDOT samples reveals a similar correlation between N in PEDOT and NO_3_^−^ in solution (Fig. 3b). The lower limit of N uptake into the PEDOT is observed to occur between 0.02 and 0.2 ppm NO_3_^−^ concentration. At 0.02 ppm it cannot be concluded that N is present in the PEDOT merely that it is below the measurement resolution of the XPS. The lower limit is demonstrated however by the measured change in sheet resistance of the PEDOT from its reduced state and after exposure to NO_3_^−^ (Fig. 3c). The percentage change for the 0.02 and 0.2 ppm NO_3_^−^ exposure is comparable. When comparing the absolute uptake of N in PEDOT from the agricultural soil water samples and the NO_3_^−^ samples, the NO_3_^−^ samples uptake more N. This is rationalised as being due to the presence of only one cation, Na^+^, in solution at the same concentration as the NO_3_^−^. Conversely the extracted agricultural water has a complex mix of anions and cations, in most cases at concentrations exceeding that of the NO_3_^−^. The complex mix of ions is hypothesised to retard the uptake of N into PEDOT.

It is necessary to compare the sensitivity of the PEDOT material for NO_3_^−^ with other more commonly used techniques, such as IC which is generally used as the benchmark technique for determining ion concentration(s). This technique has a typical minimum measurable concentration of 0.05 ppm. Otterpohl and co-workers^29^ compare a range of techniques to measure NO_3_^−^ in the presence of high concentrations of Cl^−^. The reported minimum measurable concentration across those studied was between 0.2 and 2 ppm. An electrochemical process was employed by Badea *et al*. using coated platinum electrodes^30^. While the minimum [NO_3_^−^] was not reported, concentrations of 4 to 44 ppm NO_3_^−^ was measured in mixed ion solutions. Wood and co-workers^31^ developed a nitrate selective electrode based upon N,N,N-triallyl leucine betaine chloride in a cross-linked polymer binder. This sensor covered a concentration range of 0.47 to 16 ppm NO_3_^−^. A downside to electrochemical measurements such as these is the need for a reference electrode to be immersed in the same environment. In contrast, a PEDOT film is easily fabricated, requires no reference electrode, and demonstrates selectivity for NO_3_^−^ across a comparable concentration range to other techniques even in the presence of other anions. The hypothesised origins of this selectivity relates to the π,π-enhanced anion-π interactions described by Dawson *et al*.^32^. This is a reasonable assumption given a PEDOT film is well described as a supramolecular structure with strong π-bonding and an affinity for anions.

In summary, the conductive polymer PEDOT is observed to show specific selectivity for NO_3_^−^ uptake from aqueous solutions. The NO_3_^−^ dopes PEDOT via a passive process (not requiring external driving voltages or electric fields) in preference to other anions even if present in equal or higher concentrations in solution. In the case of neat NO_3_^−^ solutions, N is detected in PEDOT from solutions as low as 0.2 ppm NO_3_^−^. In practice the change in electrical properties of the PEDOT could be used to detect the concentration of NO_3_^−^ from solution. Such sensing is of interest in relation to aqueous environments where NO_3_^−^ is present, such as agricultural water in soil. These novel findings extend the function of PEDOT beyond its typical use as a flexible conductive and optoelectronic p-type material.

## Materials and Methods

Fe(III) tosylate was received from H. C. Starck as a 54 wt.% solution in butanol (Baytron CB 54). 3,4-Ethylenedi-oxythiophene (EDOT) monomer, lithium perchlorate, sodium nitrate and sodium chloride and the block copolymers poly(ethylene glycol−propylene glycol−ethylene glycol) (PEG-PPG-PEG) in Mw = 5800 were obtained from Sigma-Aldrich. All chemicals were used without further purification.

The conductivity was calculated using σ = (R_s_.t)^−1^, where R_s_ is the sheet resistance and t is the thickness of the PEDOT film. R_s_ was measured using a Jandel four point probe, and film thickness was measured with a Bruker DektakXT profilometer with 12.5 μm stylus under a 3 mg load. The surface chemistry of polymer films was analysed using an SPECS (SAGE, Phoibos 150-HSA) X-ray photoelectron spectroscopy (XPS) system fitted with a non-monochromated Al anode, with a power of 200 W and a base pressure of 2 × 10^−6^ Pa. Curve fitting was performed with Casa XPS (Neil Fairley, U.K.), using a linear background. Spectra were charge corrected relative to the aliphatic carbon peak at 285 eV.

PEDOT films were synthesized on cleaned quartz slides as substrate using the vacuum-VPP process. Prior to polymerisation, substrates were air plasma treated (PDC-32G, Harrick Inc.) for 2 min. The oxidant film was directly spin-coated (400B-6NPP, Laurell Technologies Inc.) onto substrate at 1500 rpm for 25 s and then placed on a hotplate set to 70 °C for 30 s to evaporate the solvents. Samples were then removed from the hotplate and placed into a polymerisation chamber (Binder Vacuum Oven-VD 115) set to 35 °C. After polymerisation, PEDOT films were carefully washed with ethanol to remove any unreacted species and existing oxidant solution. Since PEDOT is almost fully doped via the VPP process, a three electrode system in electrochemical cell was employed to electrochemically reduce the PEDOT films in the presence of a standard Ag/AgCl electrode (3.8 M KCl) as the reference electrode, in order to remove most of the anions from the PEDOT films.

Three different extracted water samples from the field were provided by Sentek Pty Ltd. Provided samples were filtered using Acrodisc Syringe Filter 0.45 μm Supor Membrane to remove particulate material, and then electrochemically reduced PEDOT films were exposed to the samples. PEDOT films were kept in the water samples for one hour, and then dried by air. Treated PEDOT films were investigated before and after treatment using various techniques, investigating the electrical and optical properties, in addition to their chemical composition.

## Electronic supplementary material

Supporting Information
