# Supplementary material for: Selective uptake and sensing of nitrate in poly(3,4-ethylenedioxythiophene)
Source: Sci Rep. 2017 Nov 29;7:16581. doi: 10.1038/s41598-017-16939-5 (PMC5707362; doi:10.1038/s41598-017-16939-5)
Supplement: Supplementary file 1 — Supporting Information [file 41598_2017_16939_MOESM1_ESM.pdf]

Supporting Information

Selective uptake and sensing of nitrate in poly(3,4-ethylenedioxythiophene)

Sam Rudd[1], Michael Dalton[2], Peter Buss[2], Amanda Treijs[2], Michael Portmann[2], Nick Ktoris[2], Drew Evans[1]*

[1]Future Industries Institute, University of South Australia, Mawson Lakes, South Australia, 5095 Australia

[2]Sentek Pty Ltd, Stepney, South Australia, 5069 Australia

*Email: drew.evans@unisa.edu.au

Figures

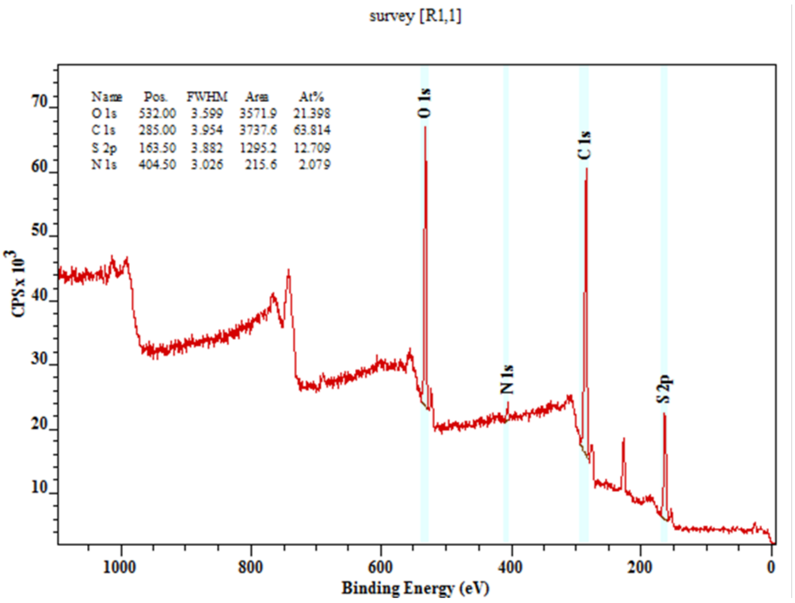

Reduced PEDOT-exposure to NO_3^- (0.5M)

Figure S1. Chemical analysis of reduced PEDOT film treated with electrolyte containing nitrate, using X-ray photoelectron spectroscopy (XPS)

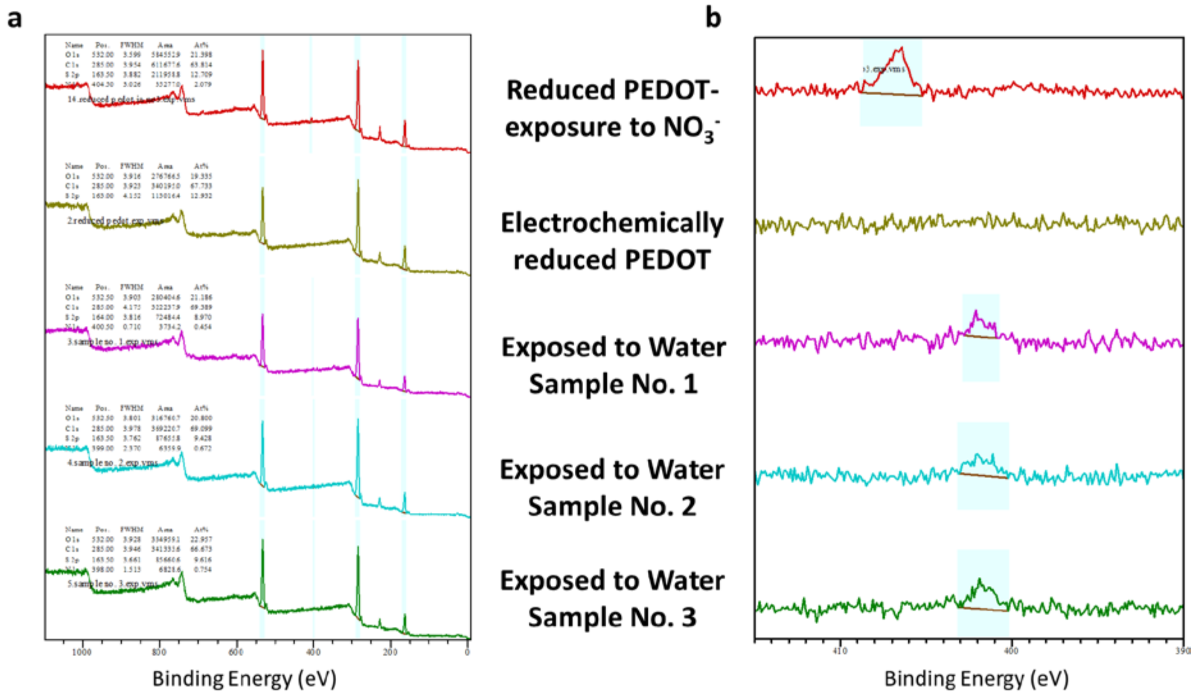

Figure S2. Chemical analysis of PEDOT films treated with extracted water samples from the field. (a) survey scan using XPS and (b) high resolution scan of N (nitrogen) in PEDOT exposed to the extracted waters, which shows a shift to the right side in comparison with salt solution of nitrate. This comparison suggests a different form of nitrate in the field.

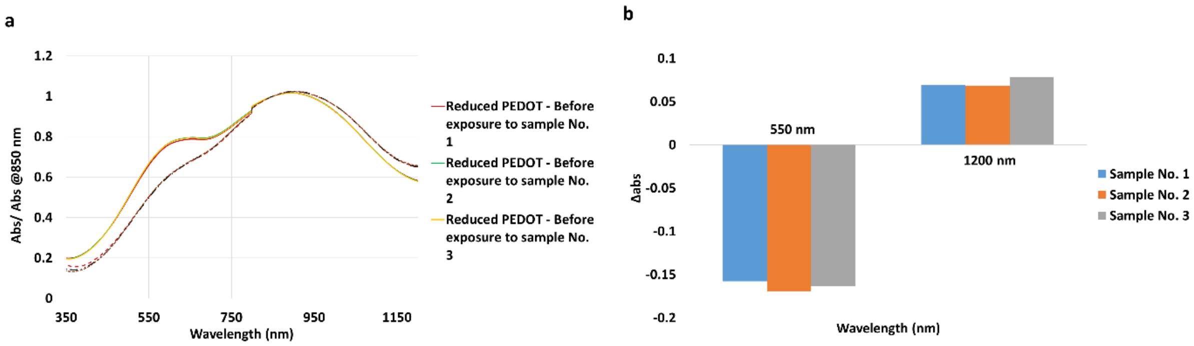

Figure S3. (a) Absorption spectra and (b) $\Delta_{absorbance}$ of reduced PEDOT before and after treatment with extracted water samples from the field.

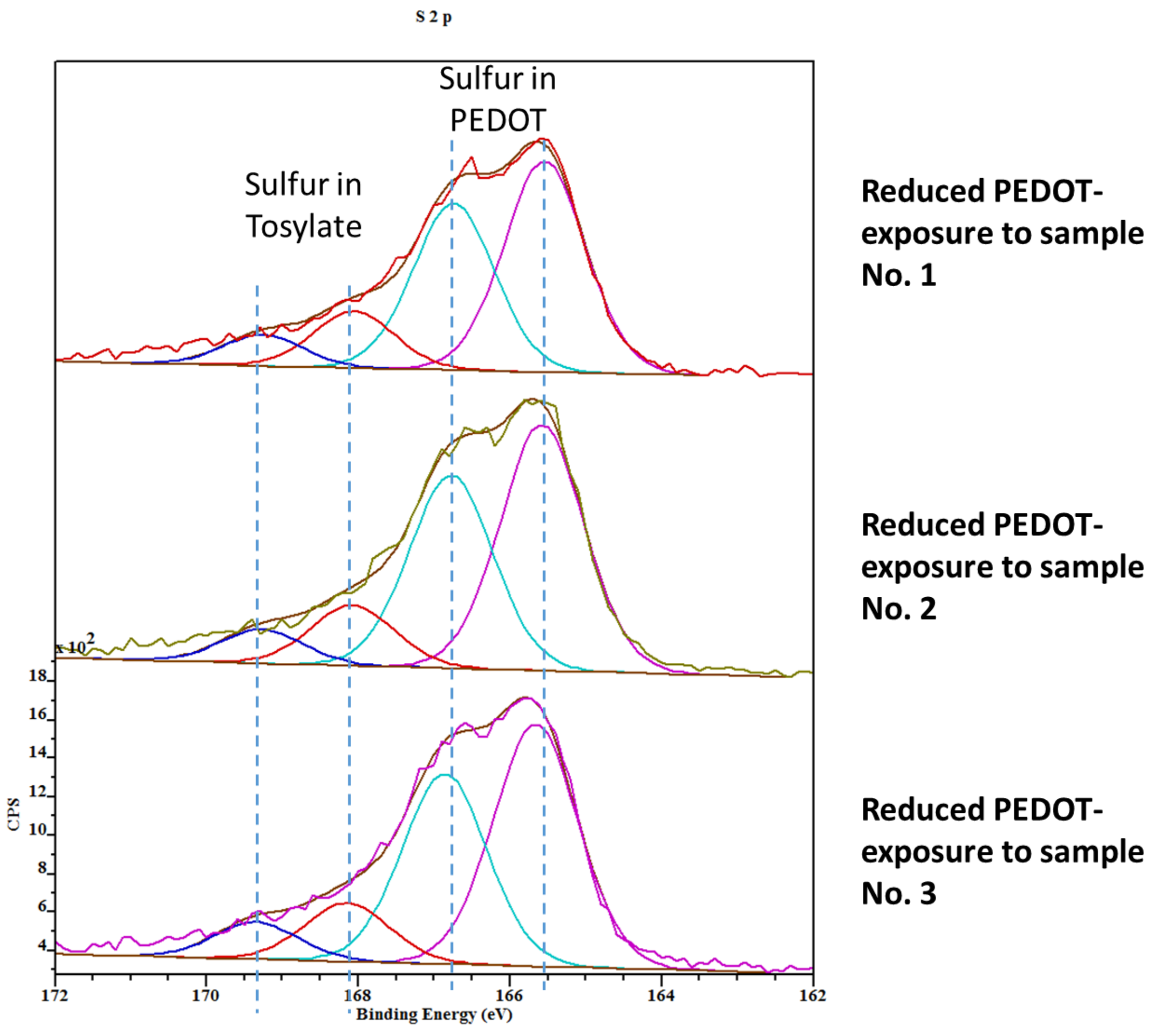

Figure S4. Analysis of the S 2p fine scan in PEDOT exposed to the extracted water samples
